# Towards Two-Photon Polymerization-Compatible Diffractive Optics for Micro-Mechanical Applications

**DOI:** 10.3390/mi14071319

**Published:** 2023-06-27

**Authors:** Victoria Paige Stinson, Uma Subash, Menelaos K. Poutous, Tino Hofmann

**Affiliations:** Department of Physics and Optical Science, University of North Carolina at Charlotte, 9201 University City Blvd., Charlotte, NC 28223, USA

**Keywords:** micro-mechanics, beam splitting, diffractive optics, two-photon polymerization, optical sensing

## Abstract

Diffractive optics are structured optical surfaces that manipulate light based on the principles of interference and diffraction. By carefully designing the diffractive optical elements, the amplitude, phase, direction, and polarization of the transmitted and reflected light can be controlled. It is well-known that the propagation of light through diffractive optics is sensitive to changes in their structural parameters. In this study, a numerical analysis is conducted to evaluate the capabilities of slanted-wire diffraction gratings to function opto-mechanically in the infrared spectral range. The slanted wire array is designed such that it is compatible with fabrication by two-photon polymerization, a direct laser-writing approach. The modeled optical and mechanical capabilities of the diffraction grating are presented. The numerical results demonstrate a high sensitivity of the diffracted light to changes in the slant angle of the wires. The compressive force by which desired slant angles may be achieved as a function of the number of wires in the grating is investigated. The ability to fabricate the presented design using two-photon polymerization is supported by the development of a prototype. The results of this study suggest that slanted-wire gratings fabricated using two-photon polymerization may be effective in applications such as tunable beam splitting and micro-mechanical sensing.

## 1. Introduction

Diffraction gratings have a wide range of applications such as holography, spectral analysis, integrated optics, quantum electronics, etc. [1]. For applications such as spectral filtering [2,3], antireflection [4], and waveguide coupling [5], diffractive gratings are used to optimize the system transmission efficiencies. Other applications such as pulse shaping, mode locking, Q switching, multiplexing, demultiplexing, spatial light modulation, and multiple-beam generation also depend on gratings [1]. While all these applications are distinct, they all exploit the same basic diffraction properties of a grating.

Slanted-wire gratings are an efficient design for light coupling into waveguides, which is essential in applications such as liquid crystal displays, virtual reality displays, and backlighting and has been studied for over a decade [6]. While these applications are in the visible regime, slanted gratings are also optimized for performance in the infrared regime for applications such as multi-mode interference (MMI) couplers [7]. Slanted gratings have advantages over traditional binary rectangular gratings as they can be operated at normal incidence, a useful feature in integrated optics, as it eases the complexity of alignment.

Slanted gratings are fabricated mainly using electron-beam lithography and reactive ion etching (RIE) processes. To etch at oblique angles, an equipotential Faraday cage is placed over the substrate resting at an oblique angle with the platten of the etching chamber [8]. Fabrication by focused ion beam etching using an alumina hard mask and iodine gas have also been reported [9]. Fabrication errors such as a deviation from the nominal design depth, fill factor, and distorted side walls are common in RIE-fabricated gratings due to shadowing effects [10].

While these approaches have advantages in their scalability, they require the use of sophisticated processes that can be costly and time consuming and are not applicable for rapid prototyping. The use of additive manufacturing to fabricate optics has seen attention in recent years [11]. The main advantage of 3D-printing processes is their ability to synthesize nearly arbitrary geometries. Two-photon polymerization has been the additive-manufacturing technique of choice for the development of optical devices for applications in the visible and infrared spectral range. Using this technique, resolutions can be attained below the diffraction limit of the light source [11]. This was first demonstrated in 2001 by S. Kawata et al., who successfully printed a bull figurine with feature sizes of 120 nm [12].

The ability of two-photon polymerization to fabricate nearly arbitrary geometries on a subwavelength scale is a powerful tool in the development of structures that manipulate light by diffraction. The applications of diffractive optics fabricated with this technique are expansive, ranging from biological applications such as fiber-optical microendoscopy [13] to wide-scope “lab on a fiber” devices for applications in chemical and temperature sensing [14].

In addition to the synthesis of optical devices, two-photon polymerization has been implemented in fabricating micro-mechanical systems [15,16,17,18]. Structures fabricated with this approach can be designed to have elastic functionality. As interest has grown in this area, studies have been conducted to characterize the mechanical properties of many two-photon polymerization-compatible resins [19,20,21,22]. These mechanical properties have been exploited to develop devices such as micro-electromechanical systems (MEMS) [16], biomaterial scaffolds [17], and magnetic micro-robots [18].

There is interest in combining optical and micro-mechanical capabilities in a single device. These devices are often termed “MOEMs”, which stands for micro-optical electromechanical systems. MOEMs have a range of applications in fields such as communication technology, medicine, and aerospace [23]. The utilization of MOEMs ranges from simple micro-mirror arrays [24] and photonic switches [25] to more sophisticated applications such as micro-spectrometers [26]. However, reports on devices with opto-mechanical capabilities fabricated by two-photon polymerization are scarce [27,28,29].

Micro-scale slanted-wire arrays have been realized with two-photon polymerization [30,31]. The slanted wires fabricated in these studies are on a scale that could function in the infrared spectral range as diffractive gratings. However, there has not yet been an investigation of slanted-wire diffractive gratings fabricated with this technique. The fabrication of slanted-wire gratings using two-photon polymerization could be an impactful next step in diffractive optics. While these off-axis gratings currently require a more sophisticated fabrication approach than axis-symmetric gratings, they pose no additional intricacies for fabrication with two-photon polymerization.

In this study, we numerically investigate the potential of combining the mechanical properties of a two-photon polymerization-compatible material (IP-Dip) with the unique functionality of slanted-wire diffraction gratings. Using a rigorous coupled-wave analysis approach, a slanted wire grating geometry is optimized to transfer power between the 0th and +1st order as a function of compression. The mechanical nature of the designed slanted wire grating is then investigated using finite element method simulations. The promising results of this numerical investigation suggest slanted-wire gratings fabricated with two-photon polymerization may be effective in applications such as micro-mechanical sensing and tunable beam splitting. The ability to realize the designed grating with two-photon polymerization is verified by the fabrication of a prototype. The quality of the prototype is determined using scanning electron microscope (SEM) imaging.

## 2. Model Design

**Figure 1:**
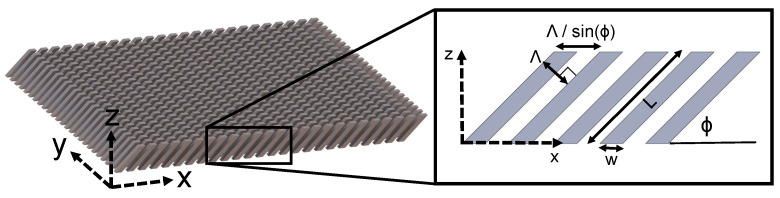
CAD model of the slanted-wire diffraction grating investigated here. During simulation, the wire width *w*, length *L*, x-axis projection of the grating period Λ/sinϕ, and slant angle ϕ were varied to optimize the design.

General diffractive concepts can be used to explain the distinct diffractive characteristics that are frequently observed in slanted-wire gratings. The slant angle is specifically selected to be close to the first Bragg angle, to increase the first diffracted order efficiency [32]. The structure parameters of the grating were designed such that only the 0th and +1st diffracted orders will be able to propagate under normal incidence conditions. This follows directly from the diffraction equation:

(1) $$\begin{array}{c} \begin{array}{c} n_{t}\sin\theta_{tm}-n_{i}\sin\theta_{i}\;=\;m\left(\frac{\lambda }{\Lambda }\right)\sin\phi ,\\ m\;=\;0,\pm 1,\pm 2,\pm 3\ldots \end{array} \end{array}$$

where $n_{i}$ and $n_{t}$ are the indices of refraction of the incident and transmitted directions, respectively. Λ and ϕ are the period and slant angle, respectively, as seen in Figure 1, and $\theta_{tm}$ and $\theta_{i}$ are the diffracted angle for the mth order and the incident angle, respectively. As the ratio λsinϕ/Λ approaches 1, the value of orders *m* for which the diffracted angle is real approaches zero (Equation (1)), indicating that the diffracted orders become progressively evanescent. Light propagates through the grating medium only for diffracted orders resulting from real angle values. This relationship can be exploited to effectively limit the number of propagating orders to two. This can be achieved by intentionally designing the grating period Λ such that it closely matches the operating wavelength λ.

Rigorous coupled-wave analysis was used in combination with the diffraction equation to calculate the diffraction efficiency, as well as the diffraction angles $\theta_{tm}$ [1]. The investigated slant angle range was restricted from 35° to 45° in order to minimize the possible inaccuracies that may result from deformations in the wire geometry during compression.

The material that was selected to design the grating for these simulations was a two-photon polymerization-compatible resin (IP-Dip). This resin provides transparency bands in the infrared and allows nanoscale resolution using two-photon polymerization [33,34]. The optical properties of IP-Dip that are used in this model have been previously determined using spectroscopic ellipsometry and are described in detail in Ref. [33]. As a starting configuration, the geometric structure parameters were adapted from a previous investigation of slanted-wire arrays that were successfully fabricated from IP-Dip using two-photon polymerization (see Ref. [31]).

The slanted wire grating is described by several structure parameters. These parameters are shown in the inset of Figure 1. The wire width *w*, length *L*, and periodicity Λ were varied in order to optimize the transmitted diffraction pattern. The slant angle ϕ was set to 45°. The design wavelength was selected to be 4 µm. X-axis linearly polarized light at normal incidence is assumed, which follows the in-plane direction of the slanted wires, as shown in the inset of Figure 1. Fused silica is selected as the substrate due to its compatibility with two-photon polymerization fabrication. The dielectric properties of the substrate were determined using spectroscopic ellipsometry.

Following the selection of the grating parameters, mechanical analysis was conducted. The Structural Mechanics module of COMSOL Multiphysics was used for finite element method mechanical simulations of a single slanted wire. The slanted wire is assumed to be an isotropic linear elastic material. During computation, the mechanical deformation was calculated using a 6 × 6 elasticity matrix [35].

**Figure 2:**
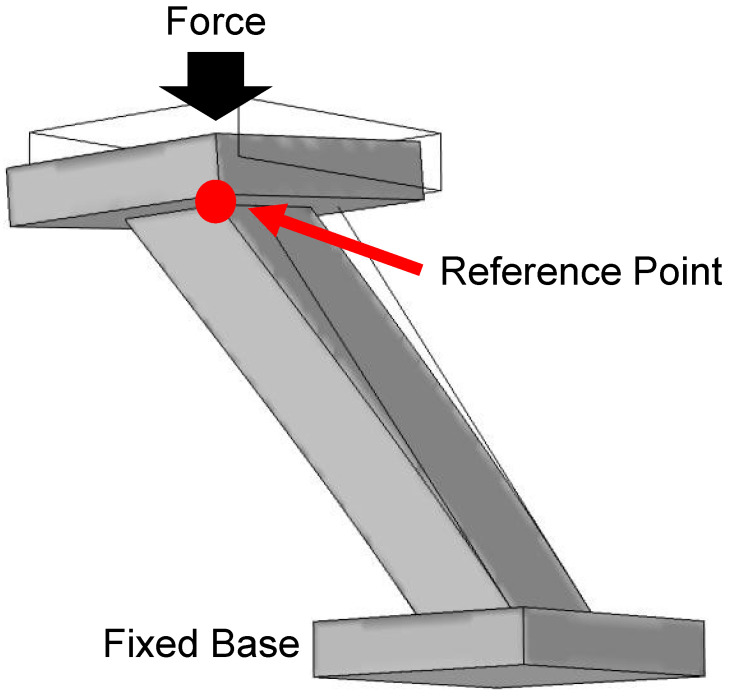
Design used for mechanical simulation in COMSOL Multiphysics. Top and bottom platforms can be seen on each end of a single slanted wire. The bottom platform is fixed while the top platform and wire are free bodies. Displacement of the slanted wire is measured with respect to a reference point on the top of the slanted wire, indicated by the red dot in the diagram.

The mechanical properties used for IP-Dip in this model were taken from Refs. [21,22]. The design used for mechanical simulation is given in Figure 2. The configuration consists of three main parts, upper and lower platforms and a single slanted wire. The platforms act as regions of attachment. The lower platform simulates the substrate on which the slanted wires are fabricated and thus, it remains fixed during simulation. The upper platform acts as the object that is compressing the slanted wire. The upper platform and slanted wire are free-moving, allowing them to displace and deform during simulation. As the applied force is varied, the z-axis displacement of the reference point on the slanted wire is monitored. This reference point is indicated by the red dot in Figure 2.

## 3. Results and Discussion

### 3.1. Optical Simulations

The slanted-wire-grating geometry was optimized such that the transmitted diffraction order efficiencies would vary as a function of the slant angle ϕ. Practically, such a variation can be achieved by compression. Since compression is proportional to changes in the slant angle, the wire width *w*, length *L*, and x-axis periodicity Λ/sinϕ were optimized using parametric sweeps to maximize the contrast in transmission efficiency as a function of the slant angle. It is worth highlighting that the diffraction orders are insensitive to changes in the y-axis periodicity. Thus, this periodicity can be selected based on fabrication constraints.

**Figure 3:**
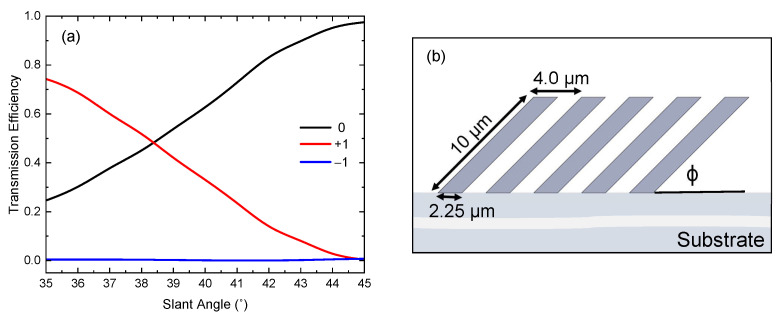
(**a**) Model calculations comparing the transmitted diffraction efficiencies for the 0th and ±1st orders as slant angle ϕ is varied. An inverse relationship is seen between the 0th (black) and +1st (red) orders while the −1st (blue) order is suppressed. (**b**) The optimized geometry used during this calculation. The values of the spatial parameters of the wire, w=2.25 µm, L=10 µm, and Λ/sinϕ=4 µm, are shown here.

The greatest contrast was found for the configuration *w* = 2.25 µm, *L* = 10 µm, and Λ/sinϕ = 4 µm. The calculated transmission efficiencies and geometry can be seen in Figure 3. As the slant angle is varied between 35° and 45°, the transmission efficiencies of the 0th (black) and +1st (red) orders share an inverse relationship while the −1st (blue) order efficiency is effectively suppressed. As the grating is compressed, resulting in a reduction in the slant angle ϕ, the power is coupled from the 0th order ($\theta_{t0}$ = 0°) to the +1st order ($\theta_{+t1}$ = 46°). For small variations in the slant angle between ϕ=45° and ϕ=35°, the power transferred from the 0th to the +1st order fluctuates from a minimum of 3% at ϕ=45° to a maximum of 70% at ϕ=35°. The power is equally shared between the 0th and +1st order at ϕ=38.4°. It is observed that the −1st order is suppressed for all slant angles within this range.

**Figure 4:**
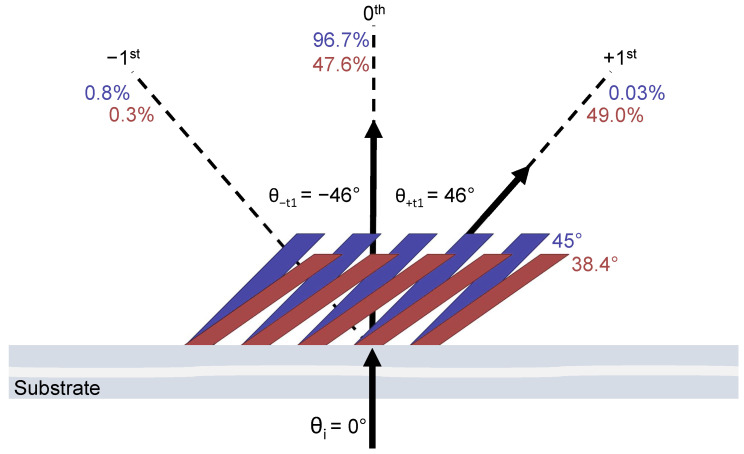
Diffraction efficiencies and diffraction angles of the 0th and ±1st orders are mapped for two slant-angle (ϕ) geometries. The efficiencies corresponding to a slant angle are color-matched. The 45° (blue) and 38.4° (red) slant-angle geometries are exaggerated for visualization. The arrows indicate the direction of propagation of the diffracted light.

The diffraction angles of the 0th and ±1st orders were investigated as a function of the slant angle ϕ. It was observed that as the slant angle was varied, the diffraction angles remained constant, as shown in Figure 4. In this diagram, two slant angles are compared. The diffraction efficiencies are provided for the different slant angles by corresponding colors. While the diffraction efficiencies vary between the 0th and +1st orders as designed, the diffraction angles do not vary.

**Figure 5:**
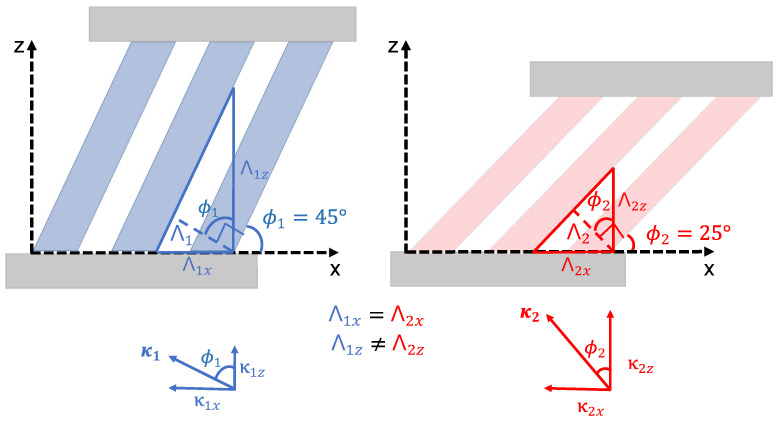
Comparison of the grating vector for two different slant angles $\phi_{1}$ and $\phi_{2}$ and their corresponding grating vector $\vec{\kappa }$. Here, the x- and z-components for $\vec{\kappa }$ and Λ are shown. While the z-components of $\vec{\kappa }$ and Λ vary for $\phi_{1}$ and $\phi_{2}$, the x-components remain constant.

The constant diffraction angles can be explained by investigating how compression changes the grating vector $\vec{\kappa }$, which results from the orientation of the grating lines in space and the spatial period, as shown in Figure 5. The direction of κ is perpendicular to the slant of the grating. Its magnitude is inversely proportional to Λ ($|\vec{\kappa }|=2\pi /\Lambda$). In Figure 5, the grating before and after compression is shown for two different slant angles $\phi_{1}$ and $\phi_{2}$ with corresponding grating vectors $\kappa_{1}$ and $\kappa_{2}$. The angle made by $\vec{\kappa }$ with the z-axis is equal to the slant angle. The x-component of $\vec{\kappa }$, which is denoted by $\kappa_{x}=2\pi \sin\phi /\Lambda$, remains constant with compression, whereas the z-component $\kappa_{z}$ varies. Since the ratio sinϕ/Λ that appears in the diffraction Equation (1) is a constant, the diffracted angles of the 0th and 1st diffracted orders in transmission are independent of the slant angle.

### 3.2. Mechanical Simulations

Finite element method simulations were conducted to characterize the mechanical capabilities of a single slanted wire. The geometry used for simulation is given in Figure 3b. The structure is defined as an isotropic linear elastic material. The mechanical properties of IP-Dip that were used during simulation were obtained from Refs. [21,22]. These mechanical parameters included Young’s modulus, Poisson’s ratio, and density and were estimated to be 2.5 GPa, 0.35, and 1200 kg/m^3^, respectively.

**Figure 6:**
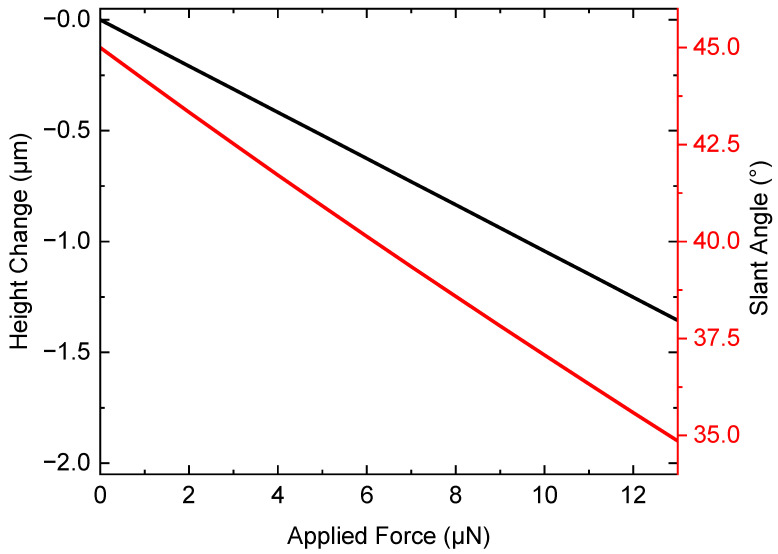
Finite element method simulated data showing the effects of applied force on the geometry of a single slanted wire are shown. The height change (black line) tracks the z-axis movement of a reference point on a single slanted wire, as shown in Figure 2. The effects on the slant angle (red line) as a function of applied force, calculated from the height change, are plotted on a second y-axis.

Figure 6 shows the results of finite element method simulations where a base and top platform are placed on a single slanted wire to act as attachment interfaces, as shown in Figure 2. The bottom surface remains fixed during simulation. A force is applied perpendicular to the top platform. The change in position of a single point is tracked as the force is varied. This reference point is shown in Figure 2. The results of the simulation are plotted in Figure 6. Here, the height change experienced by the reference point as a function of the applied force is given by the black curve. Using the calculated height change, the resulting slant angle is calculated. The slant angle as a function of the applied force is given by the red curve in Figure 6.

Based on the results of the mechanical simulation, the spring constant of a single wire is $k_{\mathrm{sw}}$ = 9.62 µN/µm. This value is calculated from the black curve in Figure 6 by taking the inverse of the slope, thereby obtaining the relationship between the applied force and displacement. To predict the stiffness of an entire array of slanted wires, the array can be treated as individual springs that are in parallel. In parallel, each spring shares the applied force. Applying Hooke’s law, the effective spring constant $k_{\mathrm{eff}}$ is then:

(2) $$\begin{array}{ccc} k_{\mathrm{eff}} & = & pk_{sw}, \end{array}$$

where *p* is the number of slanted wires in an array. Using the effective spring constant for a given array size, the degree of compression experienced by the array as a function of the applied force can be calculated using Hooke’s Law [36]:

(3) $$\begin{array}{ccc} F & = & k_{\mathrm{eff}}a, \end{array}$$

where *a* is the displacement of the slanted wire in the z-direction. The force necessary to reach a desired slant angle for an array of slanted wires can be approximated by:

(4) $$\begin{array}{ccc} F(\phi ) & = & k_{\mathrm{eff}}L\left[\sin\left(\phi_{\circ }\right)-\sin\left(\phi \right)\right], \end{array}$$

where *L* is the length of a single wire, $\phi_{\circ }$ is the fabricated slant angle, and ϕ is the slant angle desired. $k_{\mathrm{eff}}$ increases linearly with *p* (Equation (2)); thus, a linear relationship can be expected between *p* and the applied force needed to achieve a desired slant angle.

**Figure 7:**
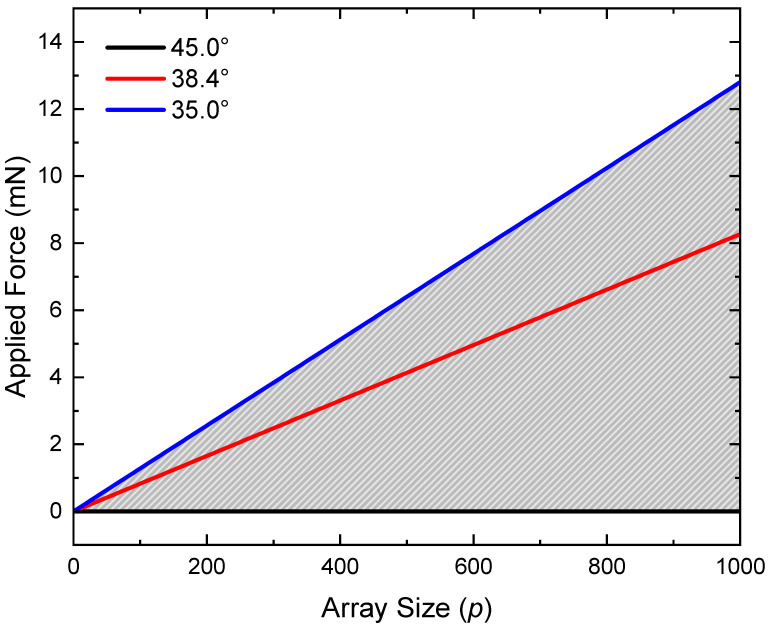
Effect of increasing array size *p* on the applied force necessary to achieve different wire slant angles. Three slant angles are plotted: 45° (black), 38.4° (red), and 35° (blue). The grey-lined region indicates the force range over which the slanted wire array can function as designed.

An analysis of this effect using the geometry given in Figure 3b is shown in Figure 7. Here, the force range over which the slanted wires will function such that the slant angle ϕ is varied between 45° (black) and 35° (blue) is plotted with respect to the array size *p*. The grey-lined region indicates the force needed to vary the slant angle between 45° and 35° as a function of *p*. The range is limited by the force required to induce a compression resulting in a slant angle of 35° (blue).

### 3.3. Fabrication Using Two-Photon Polymerization

A prototype of the designed slanted-wire grating was fabricated using two-photon polymerization. A geometry was selected such that the wire width and periodicity would be near that of the grating, as shown in Figure 3b. A commercial two-photon polymerization system (Photonic Professional GT, Nanoscribe, GmbH, Karlsruhe, Germany) was used to polymerize the design from the photosensitive resin IP-Dip. A 63× objective was used to write the structures. The best print settings were determined to be at 40% laser power (maximum 25 kW) with a 500 mm/s scan speed. The slicing and hatching distances, used to define the space between consecutive scans, were both set to 0.2 µm.

**Figure 8:**
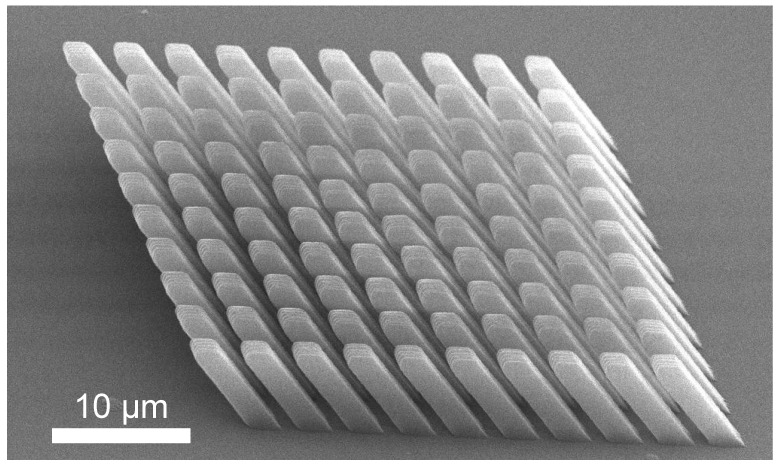
SEM micrograph of a prototype slanted-wire diffraction grating with dimensions matching the design in Figure 3b. The wires are arranged in a square lattice pattern. The wires are fabricated with high fidelity, demonstrating that mechanically modulatable three-dimensional grating structures can be obtained using two-photon polymerization.

An SEM micrograph of the resulting structure is given in Figure 8. Here, a 10 × 10 array of slanted wires can be seen with wire dimensions and lattice periodicity near that of the nominal design provided in Figure 3b. The quality of the wire structures appears to be uniform across the array, which fills a 36 × 36 µm^2^ area.

## 4. Conclusions

A numerical investigation of slanted-wire gratings compatible with fabrication by two-photon polymerization was conducted. The optical and mechanical characteristics of the designed grating were evaluated. The grating material used to conduct this analysis was a two-photon polymerization-compatible resin IP-Dip. Using previously reported optical and mechanical properties for this resin, the grating geometry was optimized parametrically to maximize the sensitivity to changes in the slant angle. A slanted-wire grating prototype was fabricated with two-photon polymerization using the optimized grating geometry.

As the grating slant angle ϕ is changed under compression between 45° and 35°, the power transitions from the 0th order to the +1st order. It is observed that the −1st order is suppressed for all slant angles within this range. The maximum power transfer is observed at ϕ=35°, while the power is almost equally shared at ϕ=38.4°. The diffraction angles of the propagating orders are found to be constant as the slant angle is adjusted. The diffraction efficiency’s sensitivity to small changes in the slant angle, while maintaining the direction of propagation, could be valuable for applications such as tunable beam splitting.

In order to evaluate the compressive functionality of the designed grating, the mechanical properties of a single wire were considered. The initial slant angle for the wire was designed to be 45°. Force was applied to the top-most surface of the wire along the z-axis to simulate the grating under compression. The change in height was monitored as a function of the applied force. This displacement was in turn used to calculate the slant angle as a function of the applied force. The spring constant of a single wire was calculated to be 9.62 µm/µN and is comparable to experimentally realized values [37].

Applying Hooke’s law to springs in parallel, the force range over which the slanted wire grating can expect to operate as a function of the array size was determined. The force required to attain a slant angle of 35° increases at a rate of 12.8 µN/wire. Increasing the array size effectively decreases the grating’s sensitivity to the applied force, which can be exploited to optimize the grating’s functionality over a desired compressive force range. By monitoring fluctuations in either of the propagating diffractive orders, the degree of compression can be determined. These results suggest that the grating may be appropriate for applications in micro-mechanical sensing. An advantage of this sensor would be in the ability to detect mechanical changes without needing physical access to the grating.

To verify the ability to fabricate the slanted-wire grating described in this study using two-photon polymerization, a prototype was developed. The geometry for this prototype was selected to match the optimized geometry presented. The quality of the fabricated array was determined using scanning electron microscopy. It is observed that the fabricated array geometry is near that of the nominal design. The quality of the slanted wires appears to be uniform across the array.

In summary, a numerical study of slanted-wire diffraction gratings compatible with two-photon polymerization was conducted. It is observed that the propagating diffraction orders of the designed grating are extremely sensitive to small changes in the slant angle. This feature, coupled with the mechanical properties the two-photon polymerization-compatible material IP-Dip, supports the use of such a grating in applications such as tunable beam splitting and micro-mechanical sensing. By varying the constituent geometry and array size, the grating can be optimized to function over a range of wavelengths and compressive forces. 

## Data Availability

The data presented in this study are available on request from the corresponding author.
